# A Diagnostic Challenge Involving Enterococcus faecalis Bacteremia: A Case Report

**DOI:** 10.7759/cureus.114988

**Published:** 2026-08-22

**Authors:** Anvitha Kambham, Maria P Farez Ochoa, Himanshukumar Nayak, Diptee Poudel, Roxana Lazarescu

**Affiliations:** 1 Medicine, Touro College of Osteopathic Medicine, New York, USA; 2 Biology, Nova Southeastern University, Davie, USA; 3 Internal Medicine/Surgery, Saba University School of Medicine, The Bottom, BES; 4 Internal Medicine, Wyckoff Heights Medical Center, New York, USA

**Keywords:** colorectal polyp, enterococcus faecalis (e. faecalis), gram positive bacteremia, pacemaker, transthoracic and transesophageal echocardiography

## Abstract

*Enterococcus faecalis* bacteremia presents a diagnostic challenge, particularly in elderly patients with multiple comorbidities and cardiac implantable electronic devices. We present the case of an 88-year-old woman with atrial fibrillation on anticoagulation, pacemaker placement, hypertension, hypothyroidism, and anxiety who presented with five days of abdominal pain, nausea, vomiting, and upper back/right shoulder pain. Initial evaluation raised concern for pneumonia due to the patient's chest X-ray and elevated temperature, and she was admitted for further management. Blood cultures at 48 hours grew Gram-positive cocci in pairs and chains and *Enterococcus faecalis*, prompting treatment with ceftriaxone and ampicillin and an extensive search for an infectious source. Transthoracic and transesophageal echocardiography showed no evidence of infective endocarditis or pacemaker-associated infection. Abdominal imaging was unrevealing. During hospitalization, the patient developed a declining hemoglobin and reported melena, prompting gastrointestinal evaluation. Colonoscopy revealed multiple colonic polyps, including tubular adenomas in the cecum and rectum, without evidence of malignancy. These adenomas were considered the most plausible portal of entry for bacteremia. Repeat blood cultures cleared, and the patient was transitioned to oral amoxicillin on discharge with outpatient follow-up. This case highlights the importance of considering gastrointestinal sources, including colorectal neoplasia, in patients with *E. faecalis* bacteremia of unclear origin. It also supports the role of multidisciplinary evaluation, including infectious disease, cardiology, and gastroenterology, in guiding workup and management.

## Introduction

*Enterococcus faecalis (E. faecalis)* is a Gram-positive, facultatively anaerobic bacterium [1]. It works as a part of the healthy human microbiome by producing vitamins, metabolizing nutrients, and maintaining intestinal pH [1,2]. It is also present in the oral cavity, genitourinary tract, especially the vaginal tract, oral cavity, and skin [1]. When there is loss of beneficial microorganisms in the microbiome, an increase in harmful pathogens, and a decrease in microbial diversity,* E. faecalis* can become pathogenic [3]. It is a common nosocomial pathogen that is implicated in infections of the gastrointestinal (GI) tract, urinary tract, wound epithelium, endocarditis, and bacteremia [2,4]. Research has also shown a prevalence of* E. faecalis* bloodstream infections in people with colorectal cancer. Although more studies on this association should be performed, colorectal cancer screening could be beneficial in those with *E. faecalis* bloodstream infection without an identified source [5]. In patients presenting with *E. faecalis* bloodstream infection, multiple potential sources of infection should be investigated. Echocardiograms are highly recommended as *E. faecalis* is a well-known cause of endocarditis and heart valve disease. Prosthetic valves and cardiac implantable electronic devices (CIEDs) could be potential sources of infection [6,7]. For those with endovascular devices, venous Doppler of the catheter site to rule out infected thrombosis is also suggested [6]. Abdominal imaging to rule out intra-abdominal abscesses or collections and urinary studies for urinary tract sources can also be performed [6]. Finally, blood cultures with susceptibilities are required to target effective antibiotic treatment [6]. Workup and proper treatment are very important as *E. faecalis* carries a mortality rate of up to 20% depending on a patient’s underlying comorbidities and antimicrobial resistance [6]. Many antibiotics can be used to treat *E. faecalis*, such as beta-lactams, vancomycin, cephalosporins, and carbapenems. But due to high levels of resistance, treatment must be adjusted based on susceptibilities, and drug combinations should be used [8]. 

This case highlights the importance of a thorough diagnostic evaluation in patients with *E. faecalis* bacteremia when a clear source is not identified. Beyond evaluation for endocarditis and common GI and genitourinary sources, unexplained bacteremia may warrant consideration of underlying colorectal pathology. This case illustrates how an initially unexplained bloodstream infection can prompt investigation for less apparent sources.

## Case presentation

An 88-year-old woman with a past medical history of atrial fibrillation on apixaban, pacemaker, hypertension, hypothyroidism, and anxiety presented to the emergency department with five days of generalized abdominal pain with associated nausea and vomiting. When asked where the pain was located, the patient pointed to the epigastrium. She presented to her primary care provider for similar symptoms two days prior and was prescribed dicyclomine with no improvement. The patient also endorsed nontraumatic upper back and right shoulder pain that has been there for about five days, ever since the patient had an intense physical therapy session. 

**Figure 1:**
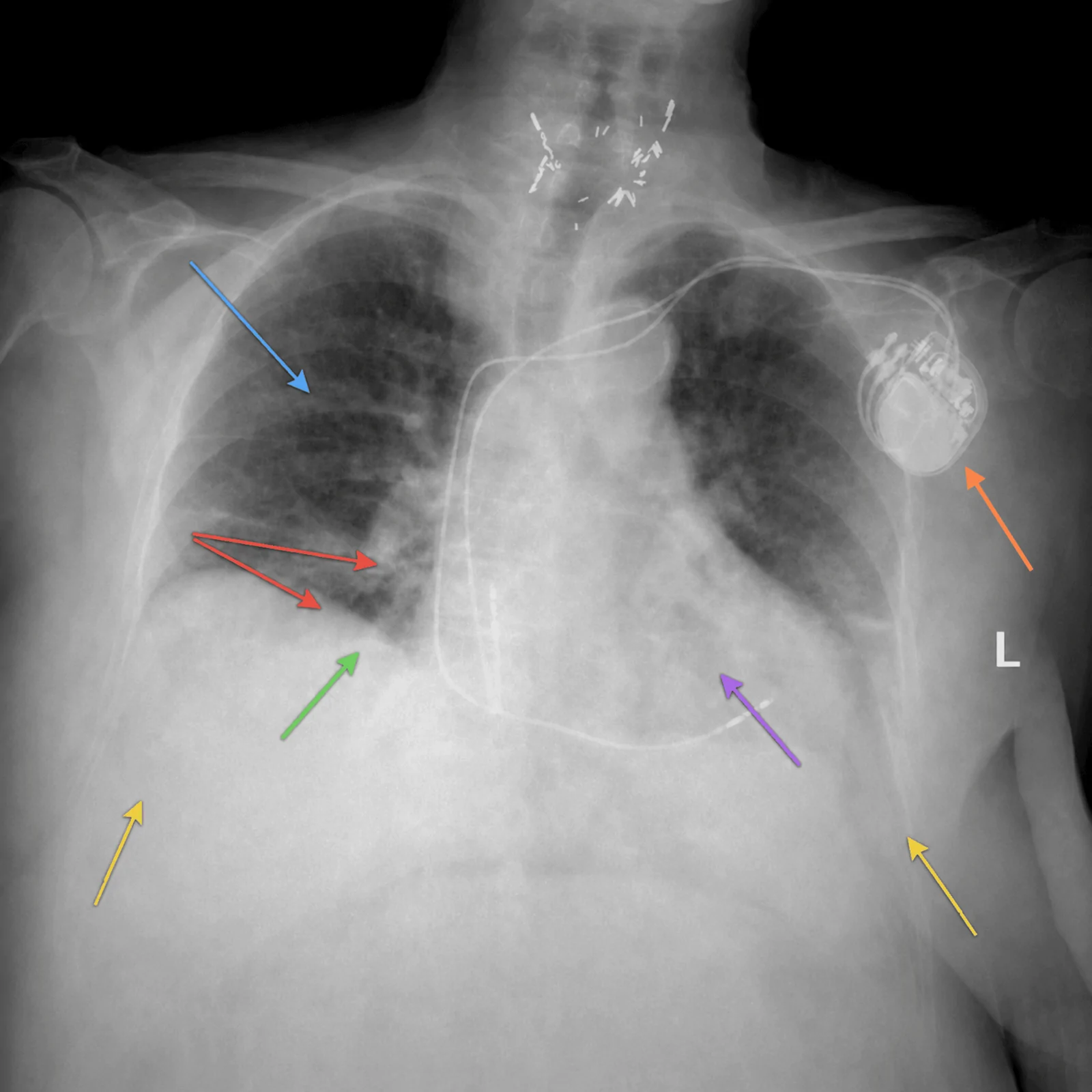
Chest X-ray X-ray of the chest showing mild pulmonary vascular congestion (blue arrow), bibasilar infiltrates (red arrow) and atelectasis (green arrow) with small bilateral pleural effusions (yellow arrow), which were questionable for pneumonia, and cardiomegaly (purple arrow) with a left-sided pacemaker (orange arrow).

In the emergency department, she had a mild temperature of 99.5 °F (37.5 °C) and was borderline bradycardic at 60 beats per minute (bpm), but other vitals were within the normal range. On repeat vitals, the patient had a temperature of 101.5°F. Although the systemic inflammatory response syndrome criteria were not met, the emergency department physician decided to start a pre-sepsis workup and ordered labs, a chest X-ray, and antibiotics (azithromycin and ceftriaxone), as well as a 500 cubic centimeter (cc) bolus of fluids to compensate for fluid lost through vomiting [9]. As shown in Figure 1, the chest X-ray showed mild pulmonary vascular congestion, bibasilar infiltrates, and atelectasis with small bilateral pleural effusions, which was questionable for pneumonia, and cardiomegaly with a left-sided pacemaker (Figure 1). Urinalysis and Adult 4-plex were negative for COVID-19, influenza A, influenza B, and respiratory syncytial virus. Electrocardiogram (EKG) showed normal sinus rhythm. Troponin was negative. Computed tomography (CT) of the abdomen and pelvis showed no acute findings and a small hiatal hernia. The patient's initial lab work on admission is shown in Table 1, displaying a neutrophil-predominant leukocytosis (Table 1). The patient was admitted due to confusion, uremia, respiratory rate, blood pressure, and an age (CURB-65) score of 2 [10].

**Table 1:** Patient’s Lab Values on Initial Presentation to the Hospital WBC, white blood cell count; RBC, red blood cell count; MCV, mean corpuscular volume; MCH, mean corpuscular hemoglobin; MCHC, mean corpuscular hemoglobin concentration; RDW, red cell distribution width; CO₂, serum bicarbonate (total carbon dioxide); AST, aspartate aminotransferase; ALT, alanine aminotransferase; GFR, estimated glomerular filtration rate; CRP, C-reactive protein.

Lab	Patient Values on Admission	Normal Range
WBC	11.5 ↑	4.5–11.0 x10³/µL
RBC Count	3.63 ↓	Male: 4.3–5.9 x10⁶/µL
Hemoglobin	11.2 ↓	Male: 13.5–17.5 g/dL
Hematocrit	33.2 ↓	Male: 41–53%
MCV	91.5	80–100 fL
MCH	30.9	26–34 pg
MCHC	33.7	32–36 g/dL
RDW	13.1	11.5–14.5%
Platelet Count	183	150–400 x10³/µL
MPV	11.3	7.5-12.0 fL
Neutrophils	71.6 ↑	54–62%
Lymphocytes	13.7 ↓	25–33%
Monocytes	13.4 ↑	3–7%
Eosinophils	0.1 ↓	1–3%
Basophils	0.2	0–0.75%
Immature Grans	1.0	0–0.5%
Procalcitonin	0.52 ↑	<0.05 ng/mL
Calcium	8.9	8.4–10.2 mg/dL
Albumin	2.8 ↓	3.5–5.0 g/dL
Total Protein	6.7	6.0–8.0 g/dL
Sodium	131 ↓	136–145 mEq/L
Potassium	2.8 ↓	3.5–5.0 mEq/L
Chloride	93	95–105 mEq/L
CO2	28	23–30 mEq/L
Blood Urea Nitrogen	39 ↑	7–18 mg/dL
Glucose	101	<140 mg/dL, non-fasting
Total Bilirubin	0.5	0.1–1.0 mg/dL
Alkaline Phosphatase	56	45–115 U/L
AST	21	10–40 U/L
ALT	25	7–56 U/L
Creatinine	1.75 ↑	Male: 0.5–1.1 mg/dL
GFR Estimation	28	>60 mL/min/1.73 m²
B-Type Natriuretic Peptide	35	<100 pg/mL
CRP, Cardio	149 ↑	<1.0 mg/L

**Figure 2:**
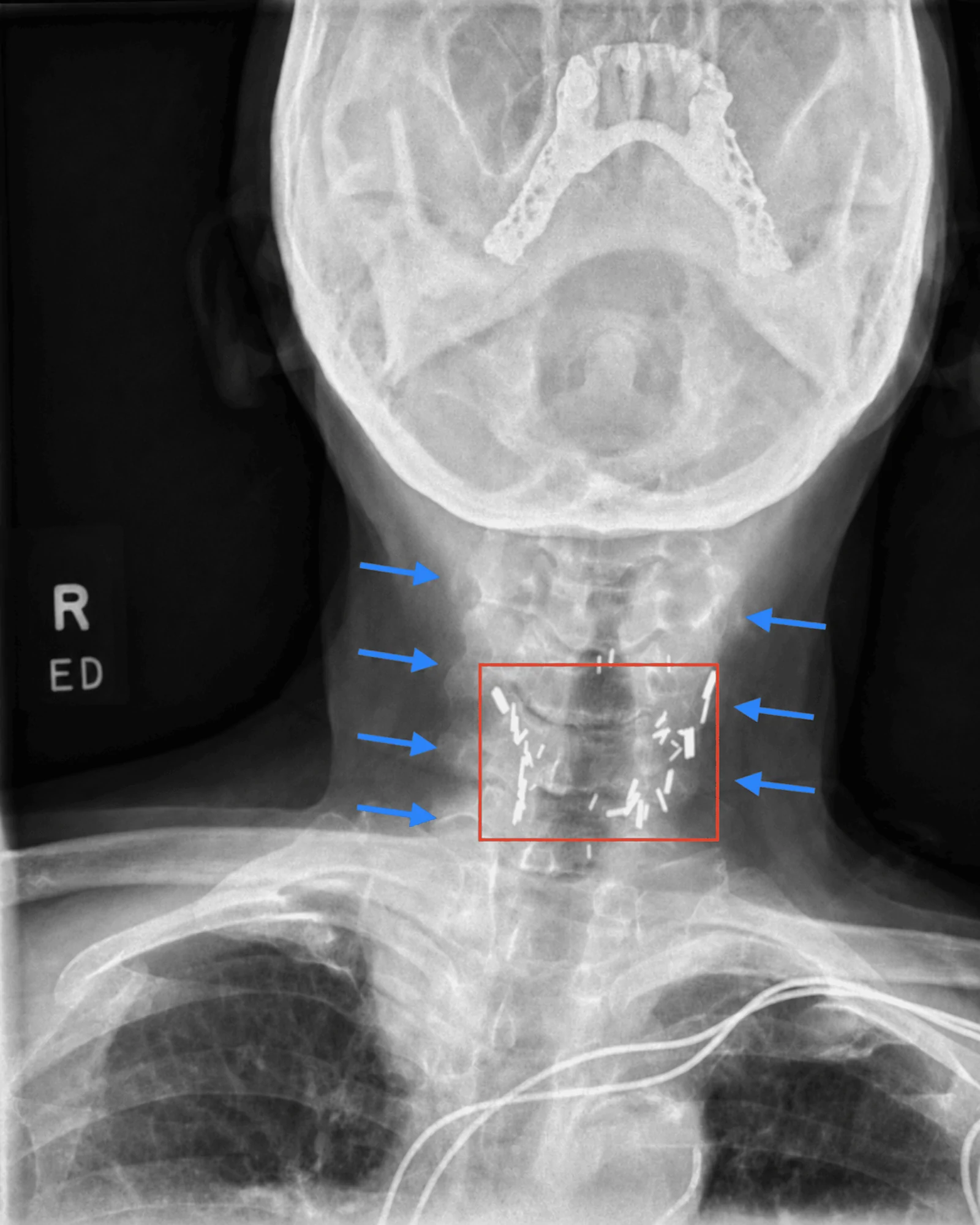
X-ray of the Cervical Spine X-ray of the cervical spine showing no fractures but moderate multilevel degenerative changes (blue arrows). Surgical clips are present in the soft tissue of the neck (red box).

**Figure 3:**
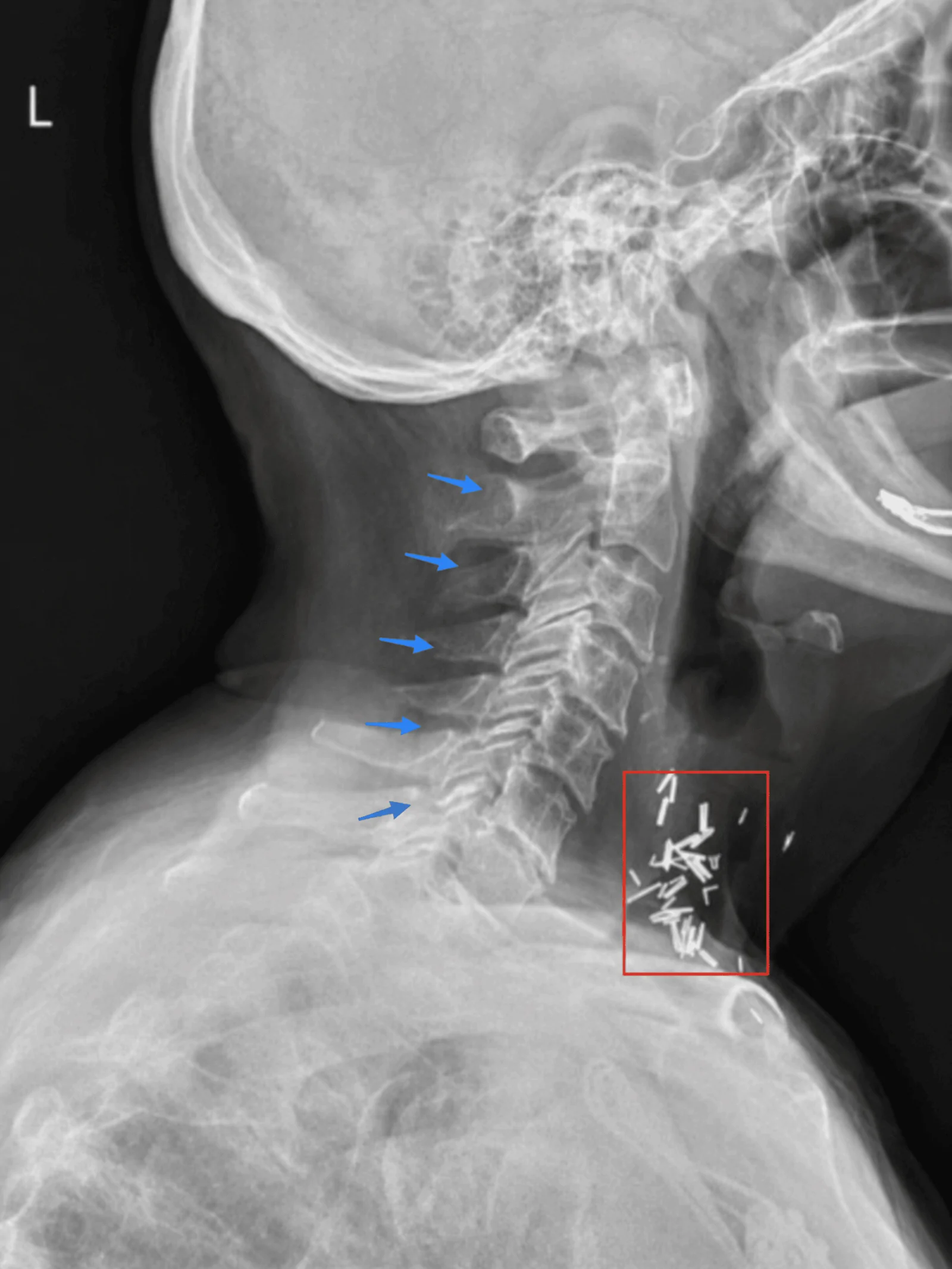
X-ray of the Cervical Spine X-ray of the cervical spine showing no fractures but moderate multilevel degenerative changes (blue arrows). Surgical clips are present in the soft tissue of the neck (red box).

**Figure 4:**
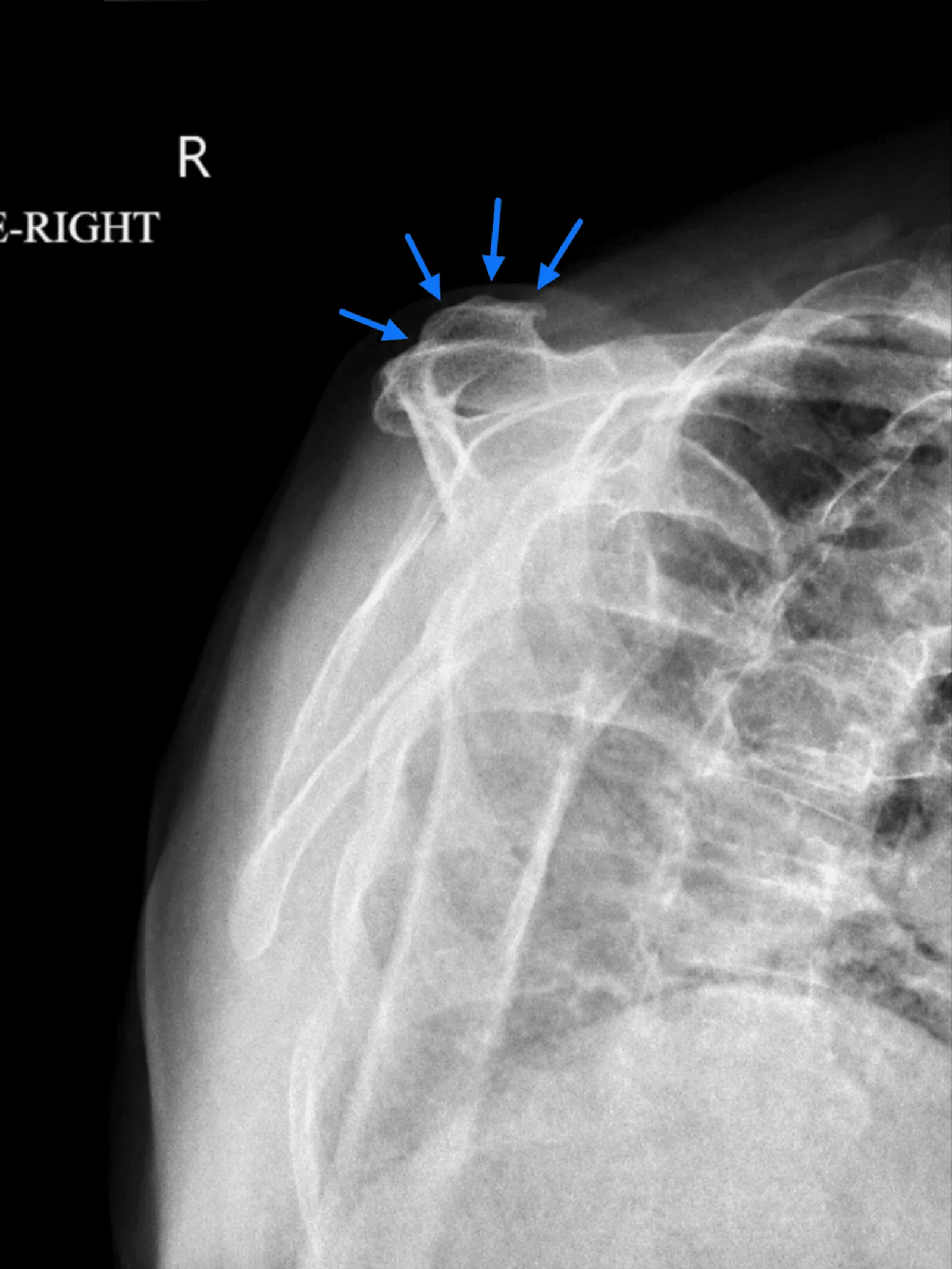
X-ray of the Right Shoulder X-ray of the right shoulder showing no acute fracture or dislocation and osteoarthritic changes at the acromioclavicular joint (blue arrows).

**Figure 5:**
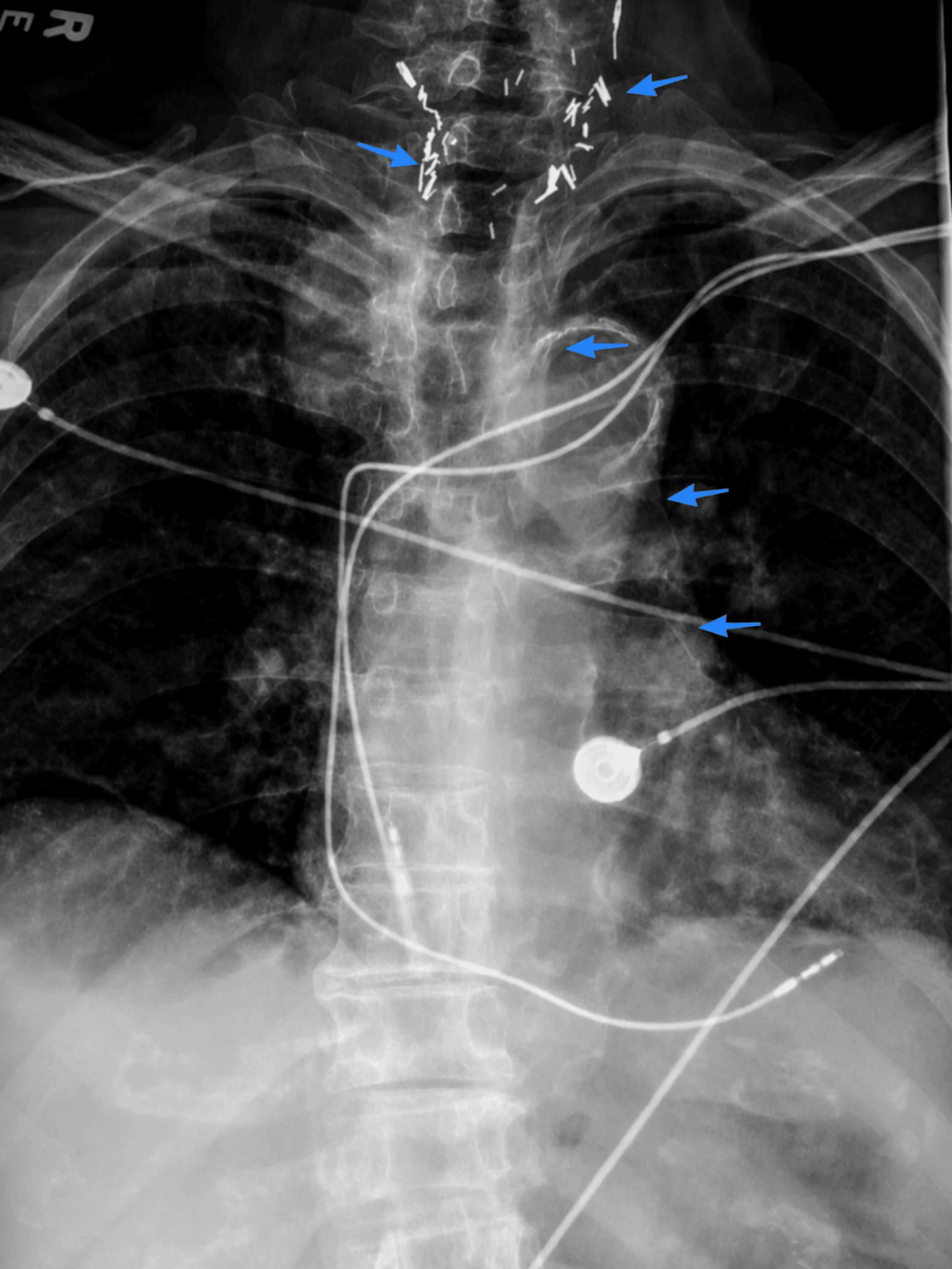
X-ray of the Thoracic Spine X-ray of the thoracic spine showing no significant abnormality of the thoracic spine and severe aortic calcifications (blue arrows).

**Figure 6:**
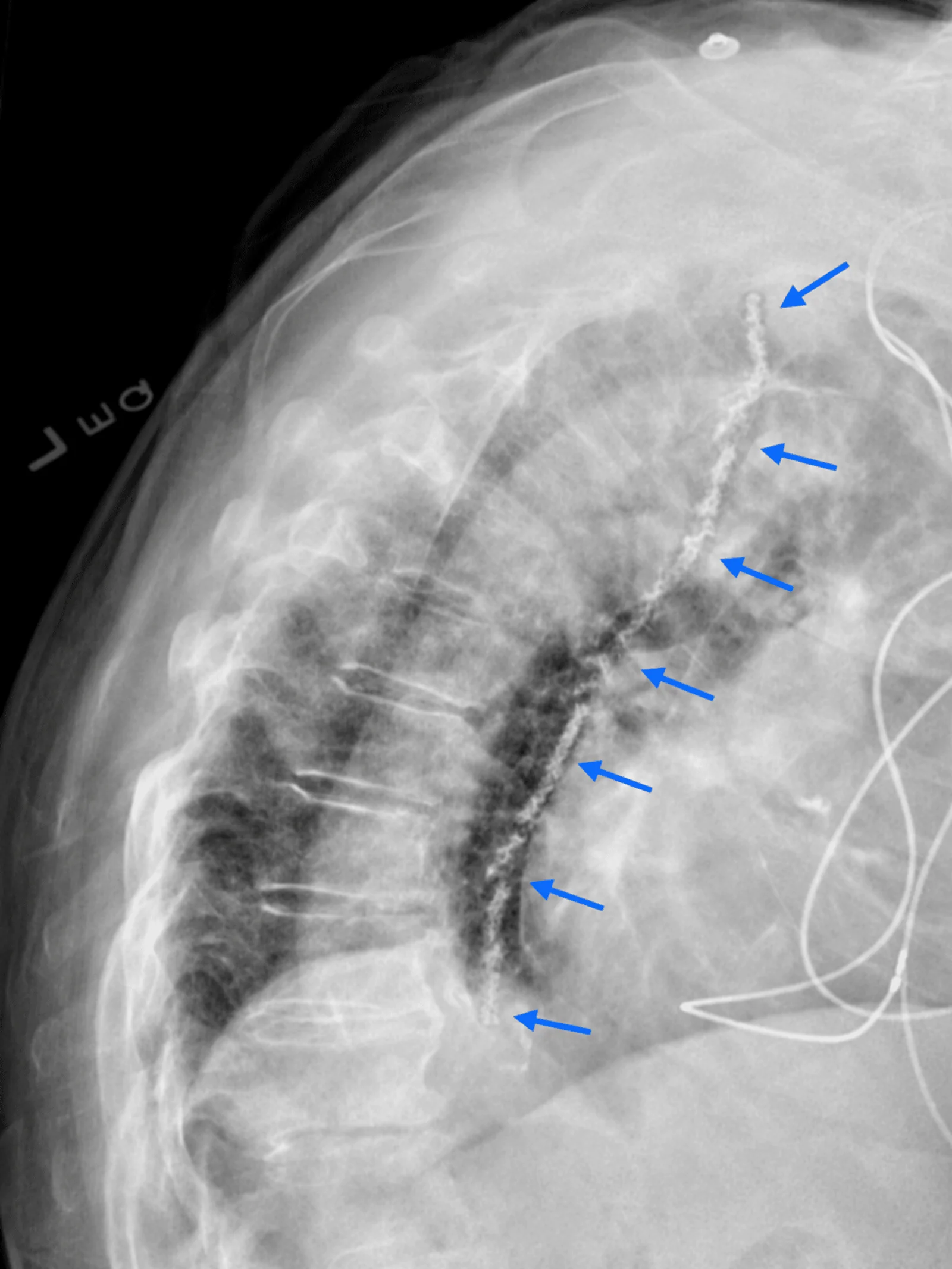
X-ray of the Thoracic Spine X-ray of the thoracic spine showing no significant abnormality of the thoracic spine and severe aortic calcifications (blue arrows).

**Figure 7:**
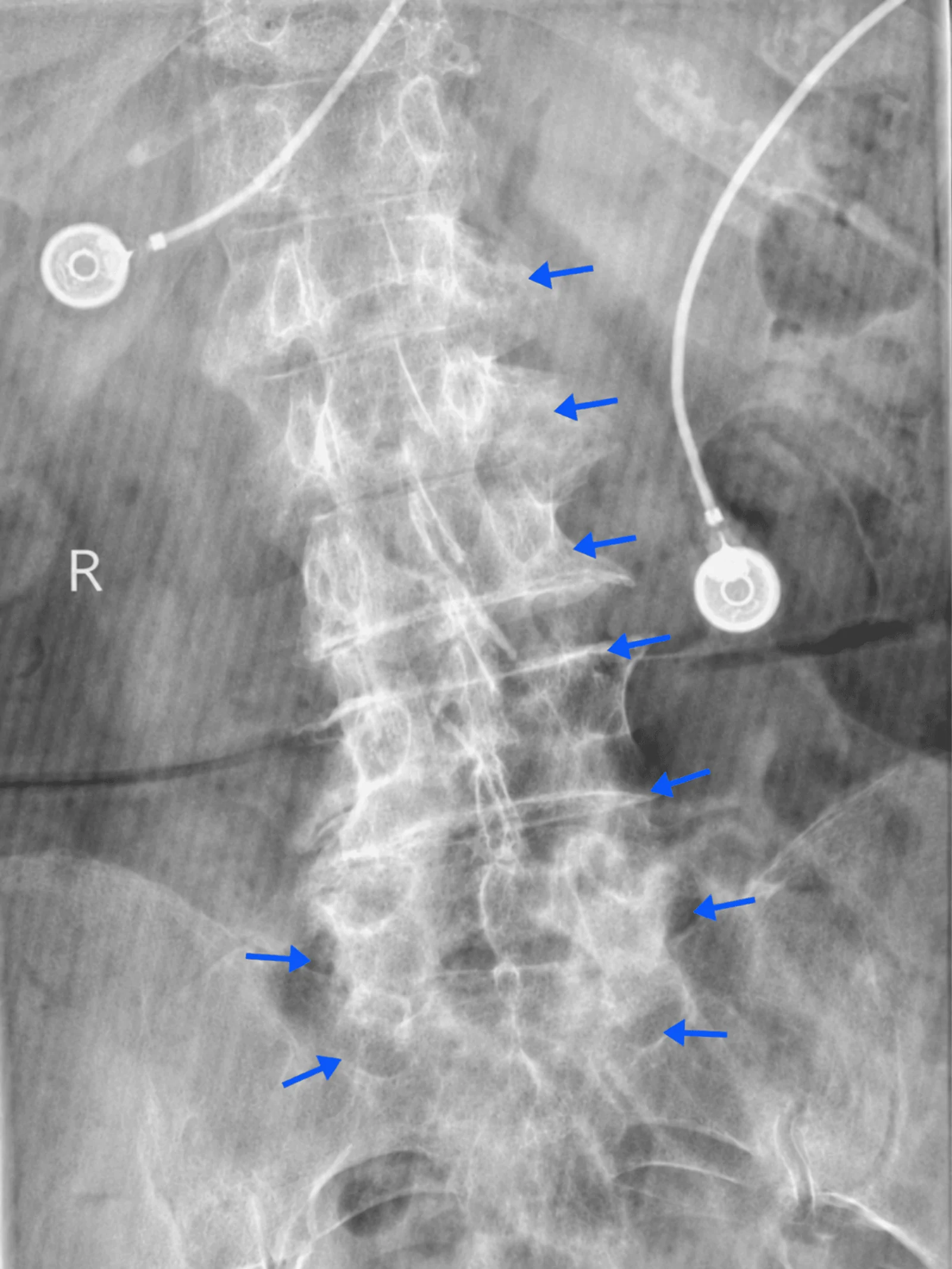
X-ray of the Lumbar Spine X-ray of the lumbar spine showing no acute fracture or subluxation and moderate multilevel degenerative changes of the lumbar spine (blue arrows).

**Figure 8:**
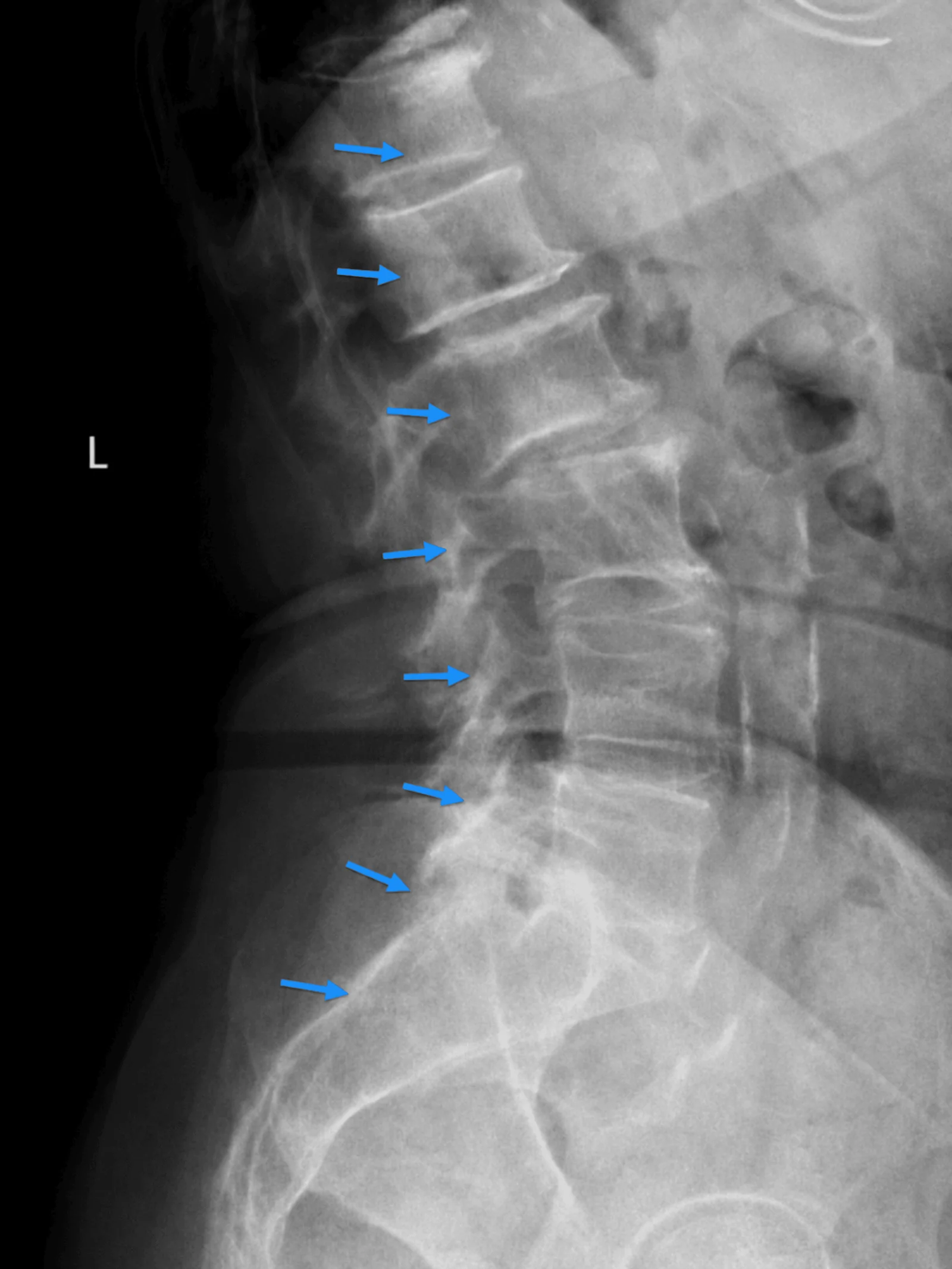
X-ray of the Lumbar Spine X-ray of the lumbar spine showing no acute fracture or subluxation and moderate multilevel degenerative changes of the lumbar spine (blue arrows).

**Figure 9:**
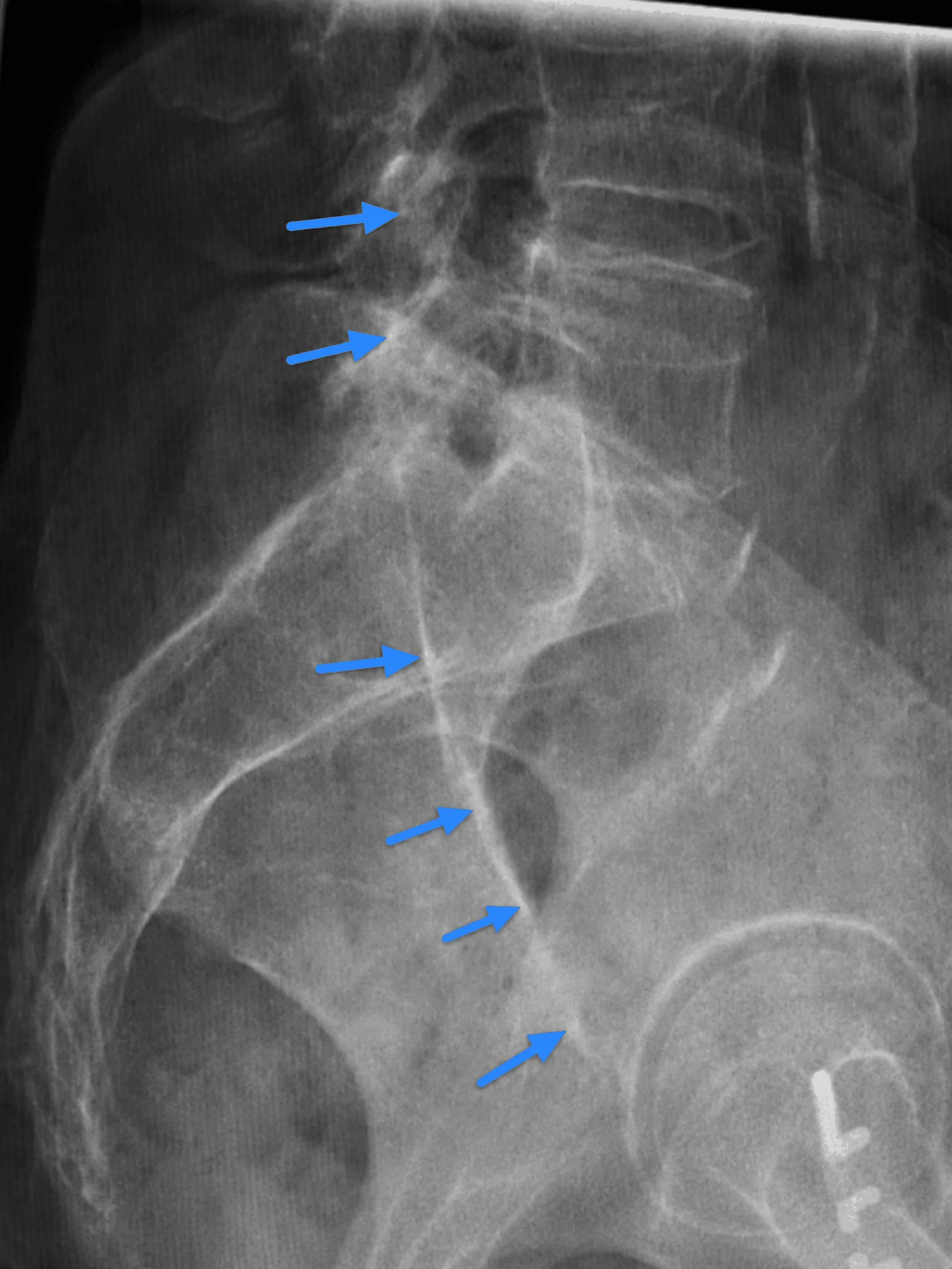
X-ray of the Lumbar Spine X-ray of the lumbar spine showing no acute fracture or subluxation and moderate multilevel degenerative changes of the lumbar spine (blue arrows).

Blood cultures, performed on hospital days two and three (at 24 and 48 hours after admission), returned positive for Gram-positive cocci in pairs and chains and *E. faecalis*. The culture showed antibiotic susceptibility to ampicillin, vancomycin, and gentamicin and resistance to streptomycin. The patient was started on ceftriaxone (2 grams (g) intravenous (IV) every 12 hours) and ampicillin (2 g IV every 4-6 hours) to cover the Gram-positive cocci in pairs and chains shown in the culture. The pneumonia workup of sputum culture, methicillin-resistant Staphylococcus aureus swab, Legionella urinary antigen, and Streptococcus pneumoniae urinary antigen test was unremarkable. Transthoracic echocardiography showed an ejection fraction of 55-60% with grade 1 diastolic dysfunction. No other acute changes were noted, and no source of infection was identified. The patient was then scheduled for transesophageal echocardiography (TEE) to investigate potential sources of infection around her pacemaker or vegetations on the heart. The TEE showed no echocardiographic evidence of infective endocarditis (IE). Tumor markers for carcinoembryonic antigen, carbohydrate antigen (CA) 19-9, CA 125, and alpha-fetoprotein were negative. To evaluate for other possible sources of infection and due to the patient’s reported pain after her physical therapy session, multiple X-rays were ordered. X-rays of the cervical spine showed moderate multilevel degenerative changes as shown in Figures 2, 3. X-rays of the right shoulder showed osteoarthritic changes at the acromioclavicular joint, as shown in Figure 4. X-rays of the thoracic spine showed no significant abnormality of the thoracic spine and severe aortic calcifications, as shown in Figures 5, 6. Finally, the X-rays of the lumbar spine showed moderate multilevel degenerative changes as shown in Figures 7-9. On hospital day 5, the patient presented with dropping hemoglobin (Hb) and self-reported two episodes of black stool. Her Hb was 11.2 g/dL on admission and 9.7 g/dL on hospital day 5. The patient states that her last colonoscopy was 6-7 years ago and was normal. Before that, it was 15 years ago, and the patient had one polyp removed and biopsied, and it came back benign. The cardiology team was consulted and decided to hold abixaban at this time. The GI team was consulted and recommended esophagogastroduodenoscopy (EGD) and colonoscopy. EGD showed mild reflux esophagitis, a 3-centimeter hiatal hernia, and gastritis erythema. Colonoscopy showed polyps; three polyps from the colon were removed and sent for biopsy. The first polyp was from the cecum and showed “fragments of tubular adenoma”. The second polyp was from the ascending colon and showed a “hyperplastic polyp”. And finally, the third polyp from the rectum showed “fragments of tubular adenoma”. There was no evidence of malignancy.

The repeat blood culture from hospital day 5 showed a negative blood culture with no growth. The duration of the bacteremia was three days. On hospital day 6, the infectious disease (ID) team was consulted and suggested discontinuing ceftriaxone (the patient was receiving this for 4 days) and continuing ampicillin (2 g IV every 4-6 hours) while the patient was admitted. Then, the patient was discharged on amoxicillin 1 g orally three times daily for four days to complete a total 10-day antibiotic course. On hospital day 8, Eliquis was resumed after no active bleeds were shown, and the patient’s Hb was stable after resuming abixaban. The patient was discharged and scheduled for follow-up in the GI clinic, ID clinic, and cardiology clinic.

## Discussion

Colonic polyps as a portal of entry

Colonic polyps are a well-recognized GI portal of entry for *E. faecalis* bacteremia. In a systematic study of IE portals of entry, GI sources accounted for 23% of cases, with colonic polyps identified in 46% of GI-related portals of entry [11]. These findings are further supported by a large Danish cohort study that demonstrated significantly elevated rates of colorectal neoplasia among patients with *E. faecalis *bloodstream infections (hazard ratio (HR) 4.64) [12], while the Grupo de Apoyo al Manejo de la Endocarditis en España (Spanish Collaboration on Endocarditis) (GAMES) cohort similarly identified colorectal neoplasia in 14.8% of *E. faecalis* IE patients [13]. In the present case, the patient's tubular adenomas in the cecum and rectum represent a plausible portal of entry, as mucosal disruption associated with adenomatous polyps can facilitate enterococcal translocation into the bloodstream.

Role of capsule endoscopy and double-balloon enteroscopy

Once a plausible source of bacteremia was identified through colonoscopy, additional small bowel evaluation with capsule endoscopy or double-balloon enteroscopy was not warranted. Several factors supported this decision: the colonic adenomas provided a sufficient explanation for the bacteremia, CT abdomen and pelvis revealed no small bowel pathology, tumor markers were negative, and hemoglobin stabilized after anticoagulation was held [14]. This approach is consistent with American Society for Gastrointestinal Endoscopy (ASGE) guidelines, which recommend capsule endoscopy only after upper and lower GI sources have been excluded [14]. These advanced modalities would be appropriate only if GI bleeding recurred following polypectomy with negative repeat bidirectional endoscopy [15].

⁸F-fluorodeoxyglucose positron emission tomography/computed tomography (¹⁸F-FDG PET/CT) as an adjunct to TEE

In this patient with a permanent pacemaker, persistent *E. faecalis* bacteremia, and negative transesophageal echocardiography, ¹⁸F-FDG PET/CT would have been a reasonable diagnostic adjunct. The 2024 American Heart Association (AHA) Scientific Statement, along with a multi-society expert consensus, rated PET/CT as "Appropriate" in patients with cardiac implantable electronic devices and fever in the setting of a negative TEE, noting that it increases diagnostic yield for occult device infection and may additionally detect extracardiac infectious foci [16,17].

Degenerative spine disease and occult vertebral infection

The patient's back pain was clinically attributed to physical therapy, and imaging evaluation was limited to plain radiographs, which have poor sensitivity for detecting early vertebral osteomyelitis. This is a notable limitation, as the Infectious Diseases Society of America (IDSA) has reported that 34% of native vertebral osteomyelitis cases are initially misdiagnosed as degenerative disease. Magnetic resonance imaging, which offers superior diagnostic accuracy with a sensitivity of 97% and a specificity of 93%, was not performed. Given the *E. faecalis* bacteremia of unclear source and the patient's degenerative spinal changes, the possibility that an early vertebral infection was obscured by pre-existing degenerative disease cannot be entirely excluded [11].

CIED management

The 2024 AHA guidelines recommend complete device removal for definite CIED infection [16]. However, a Swedish study of 72 patients with CIED and* E. faecalis* bacteremia found that when echocardiography showed no structural CIED changes, omitting extraction appeared safe, with no CIED-related recurrences [7]. These findings support device retention given the negative TEE.

Antibiotic therapy and duration

Ampicillin remains the drug of choice for susceptible *E. faecalis*. The AHA recommends ampicillin plus ceftriaxone for six weeks for enterococcal IE [18]. For uncomplicated bacteremia, a target trial emulation found no significant benefit of combination over monotherapy [19]. Regarding treatment duration, a retrospective study found short-course therapy (4-10 days) was not inferior to longer courses for uncomplicated enterococcal bacteremia (HR 1.03, 95% CI 0.65-1.62) [20]. Transition to oral amoxicillin 1 g TID for discharge was appropriate.

## Conclusions

This case illustrates the diagnostic complexity of E. faecalis bacteremia in an elderly patient with multiple comorbidities and a CIED. Despite an extensive workup, no source of infection was initially identified. Colonoscopy, prompted by declining hemoglobin and melena, ultimately revealed colonic tubular adenomas as the most plausible portal of entry, consistent with the growing literature linking E. faecalis bloodstream infections to colorectal neoplasia. This case supports the practice of routine colonoscopy in patients with E. faecalis bacteremia of unclear source, particularly in elderly patients. The patient's favorable outcome, clearance of bacteremia, negative TEE, successful polypectomy, and transition to oral amoxicillin, demonstrates the value of a multidisciplinary approach. Future prospective studies are needed to better define the optimal screening strategy for colorectal neoplasia in patients with E. faecalis bloodstream infections.
